# Development of Spinal Cord Neurons in Delicate Balance

**DOI:** 10.1371/journal.pbio.1001938

**Published:** 2014-08-26

**Authors:** Caitlin Sedwick

**Affiliations:** Freelance Science Writer, San Diego, California, United States of America

The inner cell mass of the early embryo, or blastocyst, is composed of a population of undifferentiated cells that will eventually give rise to all the tissues of the adult animal. By studying the processes that drive the differentiation of these cells, scientists hope to gain a better understanding of how these processes can go awry and glean insights into how we might counter the deficiencies and diseases that result. For instance, studying the development of spinal cord neurons could someday bring about new therapies for the treatment of spinal cord injuries or neurodegenerative diseases.

**Figure 1:**
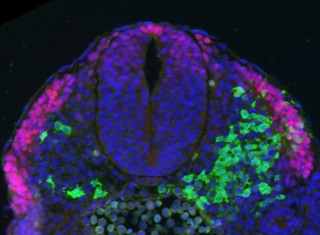
Stem cell–derived neuromesodermal precursors (green) transplanted into mouse embryos (shown in cross-section here) contribute to mesoderm, including Pax3 expressing dermomyotome (red). Tissue revealed with DAPI (blue). *Image Credit: Filip J. Wymeersch*.

Observations made in developing mouse and chicken embryos have shown that spinal cord neurons differentiate from a population of progenitor cells, neuromesodermal precursors (NMPs), located in the posterior of the developing embryo. NMPs can also differentiate into mesoderm (an early embryonic tissue that gives rise to muscle and connective tissues, along with other cell types), and accordingly, NMPs express both neural and mesodermal markers. But little else is known about NMPs because it is difficult to reach and study the region of the embryo in which these cells appear. In a collaborative study between the Briscoe and Wilson labs, published this month in *PLOS Biology*, Mina Gouti, Anestis Tsakiridis, and colleagues explore how these cells develop and demonstrate a culture system to produce NMPs in vitro.

To study NMP development, Gouti et al. employed mouse embryonic stem cells (mESCs). It is known that in vitro monolayer cultures of mESCs secrete a protein called fibroblast growth factor (Fgf) that prompts their differentiation into neural progenitor cells, a type of stem cell that is able to produce cells of the neuronal lineage. By default, these neural progenitor cells express markers typical of the forebrain, but by adding other chemicals to the cultures, researchers can prod the cells to instead display markers from different brain regions, including the hindbrain and brainstem. This culture system has therefore allowed scientists to ask questions about the development of these different brain regions that would be difficult or impossible to address in intact embryos.

Until now, the chemical signals that drive differentiation of NMPs were unknown. However, previous studies have shown that, during development, cells in the posterior of the embryo are exposed to Fgf and another secreted signaling protein called wingless (Wnt). Therefore, Gouti et al. reasoned that Fgf and Wnt may cooperate to guide development of NMPs, and could also be able to induce mESCs to differentiate into NMPs in vitro. Indeed, the authors found that if they followed the in vitro protocol to produce cells with hindbrain identity, but added in a brief, early pulse of Wnt signaling before the cells assumed neural identity, they could obtain cells expressing spinal cord–related genes.

Examination of gene expression patterns in these cells showed that they expressed both the neural marker Sox2 and the mesoderm marker Brachyury—two proteins characteristic of NMPs. Also like NMPs, the in vitro–cultured cells had a dual developmental potential. They could be prompted to differentiate into mesoderm if the Fgf and Wnt pulse was followed by exposure to Wnt in the absence of Fgf. Furthermore, when the in vitro–derived NMPs were engrafted into developing chick embryos, the descendants of these cells were found in both spinal cord– and mesoderm-derived tissues.

These data showed that the authors had successfully reconstituted in culture the conditions needed to produce NMPs. With slight adjustments, Gouti and colleagues were also able to adapt and refine their in vitro NMP culture protocol for use with other types of stem cells: mouse epiblast stem cells and human embryonic stem cells. Like their mESC counterparts, these other stem cells could be induced to take on NMP identity after exposure to Fgf and a brief pulse of Wnt, demonstrating that the developmental signals for the generation of NMPs are shared across multiple species.

Having discovered the conditions necessary to generate bona fide NMPs in vitro, Gouti et al. next took advantage of this system to investigate how Wnt and Fgf contribute to the developmental fate of embryonic spinal cord and trunk mesoderm. Ultimately, they showed that the mesoderm marker Brachyury is necessary to maintain the dual developmental potential of NMPs. In its absence, Wnt signaling was no longer able to promote mesoderm fate, and instead, cells with spinal cord identity were always formed. Together, these experiments advance our understanding of the cells that give rise to embryonic spinal cord and trunk mesoderm development.

**Gouti M, Tsakiridis A, Wymeersch FJ, Huang Y, Kleinjung J, et al. (2014) *In Vitro* Generation of Neuromesodermal Progenitors Reveals Distinct Roles for Wnt Signalling in the Specification of Spinal Cord and Paraxial Mesoderm Identity.** doi:10.1371/journal.pbio.1001937

